# HSV-1 LAT promotes ocular diseases in the absence of type I IFN responses in sensory neurons

**DOI:** 10.1128/jvi.00960-26

**Published:** 2026-08-27

**Authors:** Shaohui Wang, Ujjaldeep Jaggi, Jay J. Oh, Deepak Arya, Homayon Ghiasi

**Affiliations:** 1 Department of Surgery, Center for Neurobiology and Vaccine Development, Ophthalmology Research, Cedars-Sinai Health Sciences University 711473, Los Angeles, California, USA; University of Toronto, Toronto, Ontario, Canada

**Keywords:** IFNAR1 conditional, type I interferon, ocular infection, eye disease, virus replication, latency, reactivation, CD8, PD-1, exhaustion

## Abstract

Type I interferons (IFNs) are a critical first line of antiviral defense during herpes simplex virus type 1 (HSV-1) infection. In mice, the type I IFN family includes at least 14 IFNα subtypes and a single IFNβ, each of which signals through the common receptor, IFNαβR. We previously reported that deletion of a single IFNα subtype (IFNα2A^−/−^) alters HSV-1 latency levels following ocular infection without affecting viral reactivation. While the role of type 1 IFN in HSV-1 infection has been studied extensively, how IFN type 1 responses crosstalk with latency-associated transcript (LAT) in sensory neurons remains to be elucidated. To investigate the functional relationship between LAT and type I IFN signaling, specifically within neurons, we crossed Advillin-Cre mice with IFNAR1^flox/flox^ mice to generate sensory neuron-specific IFNAR1 knockout mice (Avil-IFNAR1^−/−^). Following HSV-1 infection, the survival rate of Avil-IFNAR1^−/−^ mice was significantly lower than that of infected wild-type mice. However, unlike global IFNAR1 deficiency, there were no significant survival differences between LAT(+) and LAT(−) viral infection in Avil-IFNAR1^−/−^ mice. During acute infection, loss of type I IFN signaling in sensory neurons enhanced viral replication in the eye and trigeminal ganglia, but not in the tears of infected mice. During latency, sensory neuron-specific IFNAR1 deficiency increased gB DNA copy numbers in LAT(−)-infected mice to levels similar to those in LAT(+)-infected mice. LAT transcript levels in these mice were also markedly higher than those in wild-type mice. The absence of neuronal type 1 IFN signaling increased T-cell exhaustion and eye disease in infected mice. However, the absence of IFNAR1 did not affect reactivation in these mice. Collectively, these findings demonstrate that neuron-intrinsic type I IFN signaling plays a critical role in controlling ocular viral replication, limiting HSV-1-induced eye diseases, and modulating latency-associated viral and immune parameters.

**IMPORTANCE:**

HSV-1 LAT plays an important role in latency reactivation, while type I interferon (IFN) regulates immune protection; the interaction of HSV-1 LAT with type I IFN influences virus infectivity *in vivo*. To investigate how LAT and IFNαβ affect the peripheral nervous system, we generated IFNAR1 conditional knockout mice using Advillin-Cre mice, which lack functional type 1 IFN signaling in the peripheral nervous system. The absence of interferon A receptor 1 (IFNAR1) in these infected mice led to enhanced viral replication in the eyes and trigeminal ganglia (TG), with increased ocular disease and T-cell exhaustion. However, viral titers in mouse tears and reactivation kinetics remained unchanged. In peripheral sensory neurons, the absence of IFNAR1 was associated with (i) similar gB DNA levels in TG of latently infected Avil-IFNAR1^−/−^ mice compared to control infected mice, (ii) LAT expression in Avil-IFNAR1^−/−^ mice was significantly elevated as compared with control infected mice, (iii) notably, IFNAR1 deficiency led to significantly more LAT-dependent eye disease in Avil-IFNAR1^−/−^-infected mice, and (iv) T-cell exhaustion was enhanced significantly in Avil-IFNAR1^−/−^-infected mice. These findings demonstrate that type I IFN signaling in sensory neurons restricts HSV-1 pathogenesis and modulates LAT expression without affecting reactivation.

## INTRODUCTION

Herpes simplex virus type 1 (HSV-1) is a double-stranded DNA virus that enters the infected host body through mucosal surfaces or broken skin and replicates at the initial site of infection, followed by retrograde transport of the viral capsid along sensory neurons to the dorsal root ganglia, where the virus becomes dormant and establishes latency in the trigeminal ganglion (TG) in the central nervous system (1–5). In the herpes eye model, exposure to external stimuli triggers HSV-1 reactivation in the TG and its retrograde transport back into the cornea via anterograde transport, causing recurrent eye infections (3, 5–7). The inflammation triggered by these recurrences can cause corneal scarring (CS), also known as herpetic stromal keratitis, which can lead to impaired vision (8–10). During latency, HSV-1 has limited transcriptional activity; transcription of lytic viral genes is minimal, and the latency-associated transcript (LAT), a long non-coding RNA, is the major viral transcript detected in infected hosts (2, 11, 12). LAT has anti-apoptotic activity, which is required to maintain latency (13). Mice and rabbits infected with LAT(−) viruses show more apoptosis, less latency, and slower reactivation than those infected with LAT(+) viruses (14–16). Replacing LAT with two different anti-apoptosis genes restores the LAT(−) virus reactivation phenotype to WT levels, indicating that anti-apoptotic LAT activity is an important component of the LAT reactivation phenotype (17–19). LAT upregulates herpesvirus entry mediator (HVEM) expression (20, 21) and downregulates type 1 interferon (IFN) responses by downregulating JAK1 and JAK2 pathways as well as downstream genes, such as ISG-15, ISG-54, ISG-56, IP-10, IRF-1, and IRF-3 (13, 22).

Interferon responses are rapidly initiated by pattern recognition receptors, such as TLR9, cGAS, and IFI16, that recognize viral DNA and proteins, thereby limiting viral replication, inducing infected cell apoptosis/autophagy, and helping to mobilize other immune responses (23–25). Many viruses have evolved strategies to evade the IFN system, such as blocking IFN induction, inhibiting downstream signaling events following cytokine-receptor binding, and inhibiting the functions of IFN-induced proteins (26–28). IFNs are divided into types I, II, and III (29). Type I IFNs’ strong antiviral activity can control viral infections in both mice and humans (24, 25). In humans, the type I IFN gene family currently includes 14 IFNα subtypes, a single IFNβ gene, and several other, less common genes (30, 31). Mice have a similar type I IFN system (32–34). All members of the type I IFN family bind to a common heterodimer receptor, composed of interferon αβ receptor 1 (IFNAR1) and interferon αβ receptor 2 (IFNAR2), which is required for their biological activities (35). IFNAR1^−/−^ mice, which lack antiproliferative and antiviral responses, are associated with IFNα/β signaling and are highly susceptible to viral infections (34, 36). Although all IFNα subtypes, as well as IFNβ, signal through the same IFNαβR receptor complex, they are not functionally identical. Differences in receptor-binding affinity, signaling kinetics, and downstream interferon-stimulated gene (ISG) induction profiles result in distinct biological outcomes (35). We previously reported that in the absence of IFNα2A, HSV-1 latency decreases (37), whereas in the absence of IFNβ, LAT(−) virus latency increases to levels similar to those in LAT(+) (37). IFNAR1^−/−^ mice are more susceptible to LAT(−) virus infection than to LAT(+) virus infection, indicating that LAT has a protective role in preventing host death from infection (36).

Since IFNAR1^−/−^ mice are highly susceptible to HSV-1 infection, we were not able to evaluate the role of type I IFN in HSV-1-induced eye disease and latency reactivation in IFNAR1^−/−^ mice (36). As neurons are the major site of HSV-1 latency, we generated a sensory neuron-specific IFNAR1 knockout mouse to study crosstalk between LAT and type 1 IFN in the primary and latent phases of infection. In Avil-IFNAR1^−/−^ mice, IFNαβ was selectively ablated in the peripheral nervous system, whereas IFNAR1^−/−^ mice lack functional IFNαβ responses globally. We found that deletion of IFNAR1 in sensory neurons caused a modest increase in mortality following HSV-1 infection but resulted in significantly higher eye disease when infected with LAT(+) virus, not LAT(−) virus. Assessment of viral infectivity during latency, as measured by gB levels, revealed no significant differences between WT and knockout mice. We also tested LAT expression in both mouse groups infected with the LAT(+) virus, which was significantly higher in Avil-IFNAR1^−/−^ mice than in WT-infected mice. Furthermore, the absence of type I IFN signaling in sensory neurons led to increased viral replication in the TG and eyes during primary infection and increased expression of exhaustion markers, including PD-1 and CD8α, during latency.

## RESULTS

### Generation of sensory neuron-specific IFNAR1^−/−^ mice

**Fig 1:**
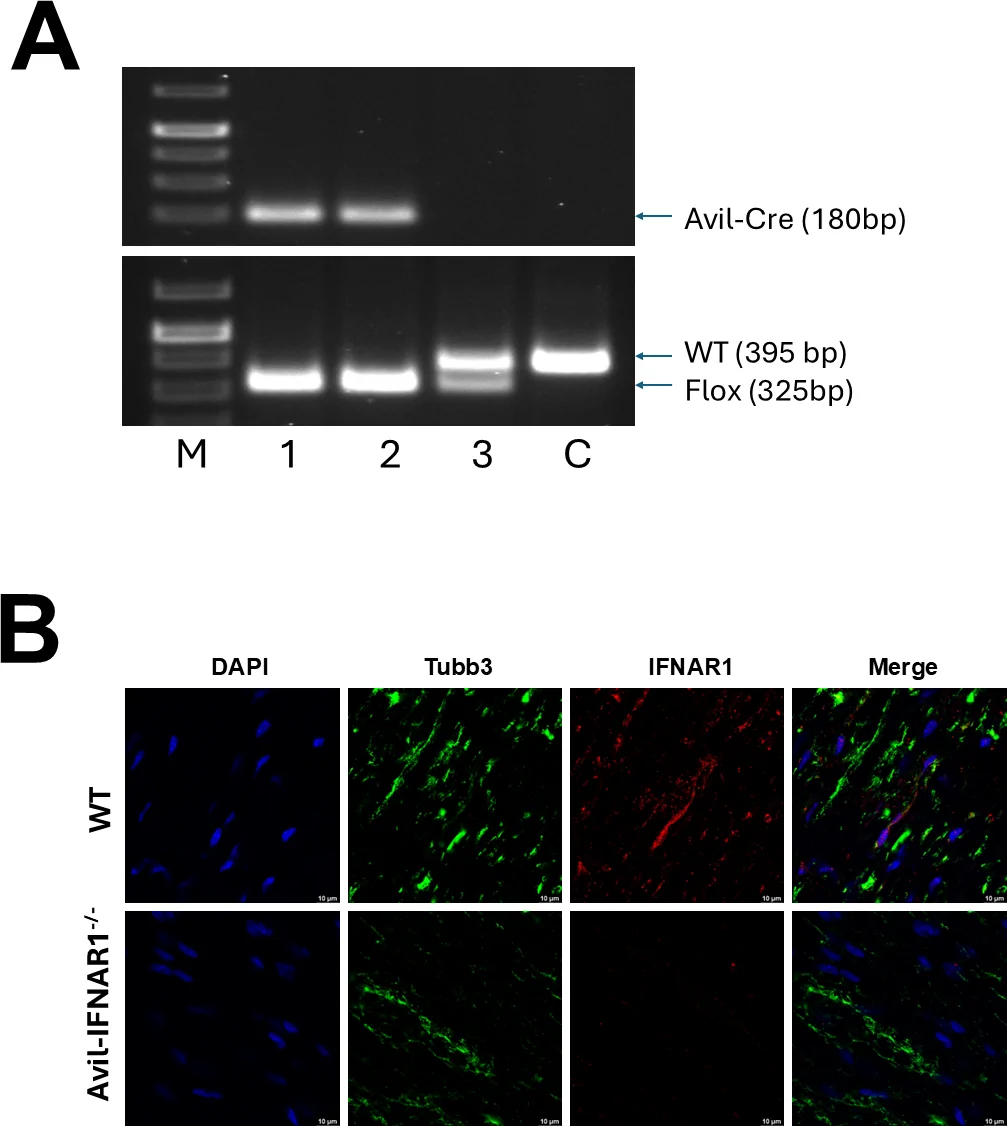
Generating and verifying sensory neuron-specific IFNAR1^−/−^ mice**.** (**A**) Generating Avil-IFNAR1^−/−^ mice. Sensory neuron-specific mice (Avil-IFNAR1^−/−^) were generated by crossing IFNAR1^flox/flox^ mice with Advillin-Cre^+^ mice to generate Avil-IFNAR1^−/−^ mice, with IFNAR1 specifically knocked out in Advillin-expressing neuronal tissue. Mouse genotypes were confirmed by PCR, as shown. After generating Avil-IFNAR1^−/−^ mice, only male Avil-IFNAR1^−/−^ mice were bred with female IFNAR1^flox/flox^ mice to generate additional Avil-IFNAR1^−/−^ mice for experimental use. (**B**) Immunostaining to verify the absence of IFNAR1 in sensory neurons. WT (top panel) or Avil-IFNAR1^−/−^ (bottom panel) TG sections were stained with anti-IFNAR1 (red) and anti-Tubb3 (green) antibodies, and the nucleus was stained with DAPI.

Type 1 IFN, which consists of multiple IFNα genes and a single IFNβ gene, is an essential antiviral cytokine that protects the host from viral infection. Knockout of IFNβ increases susceptibility to HSV-1 infection and accelerates LAT virus reactivation, while knockout of IFNα2A reduces latency and does not affect survival (37, 38). We have reported that mice with global deletion of IFNAR1 have an extremely high death rate following HSV-1 infection and that the LAT(−) virus kills all mice infected with as little as 1,000 pfu/eye (36). While the role of type 1 interferons in HSV-1 infection has been studied extensively, how interferon responses crosstalk with LAT in sensory neurons remains to be elucidated. To study the sensory neuron-specific interferon response to HSV-1 infection, we generated a conditional IFNAR1 knockout mouse in which IFNAR1 is deleted specifically in sensory neurons. As previously reported, Advillin-Cre was used to generate a sensory neuron-specific knockout (39). We generated a conditional knockout mouse line, Avil-IFNAR1^−/−^ (IFNAR1^flox/flox^ Avil-Cre^+^), by breeding IFNAR1^flox/flox^ (Jackson Laboratory #028256) mice with Avil-Cre mice (Jackson Laboratory #032536) (Fig. 1A). PCR results confirmed the IFNAR1^flox/flox^ and Avil-Cre genotype (Fig. 1A). To validate the depletion of IFNAR1 protein expression in sensory neurons, immunofluorescence staining was performed using antibodies against IFNAR1 and Tubb3 (beta-III tubulin) (Fig. 1B). The intensity of Tubb3 staining was similar in both groups, while IFNAR1 staining was markedly reduced in neurons of the Avil-IFNAR1^−/−^ group relative to the WT control (Fig. 1B). The quantification of mean fluorescence of Avil-IFNAR1^−/−^ was reduced to 22.8% compared to that in the WT group, confirming the efficient depletion of IFNAR1 expression in sensory neurons of the TG (Fig. S1).

### Effect of IFNAR1 absence in sensory neurons on virus replication in tears of infected mice

**Fig 2:**
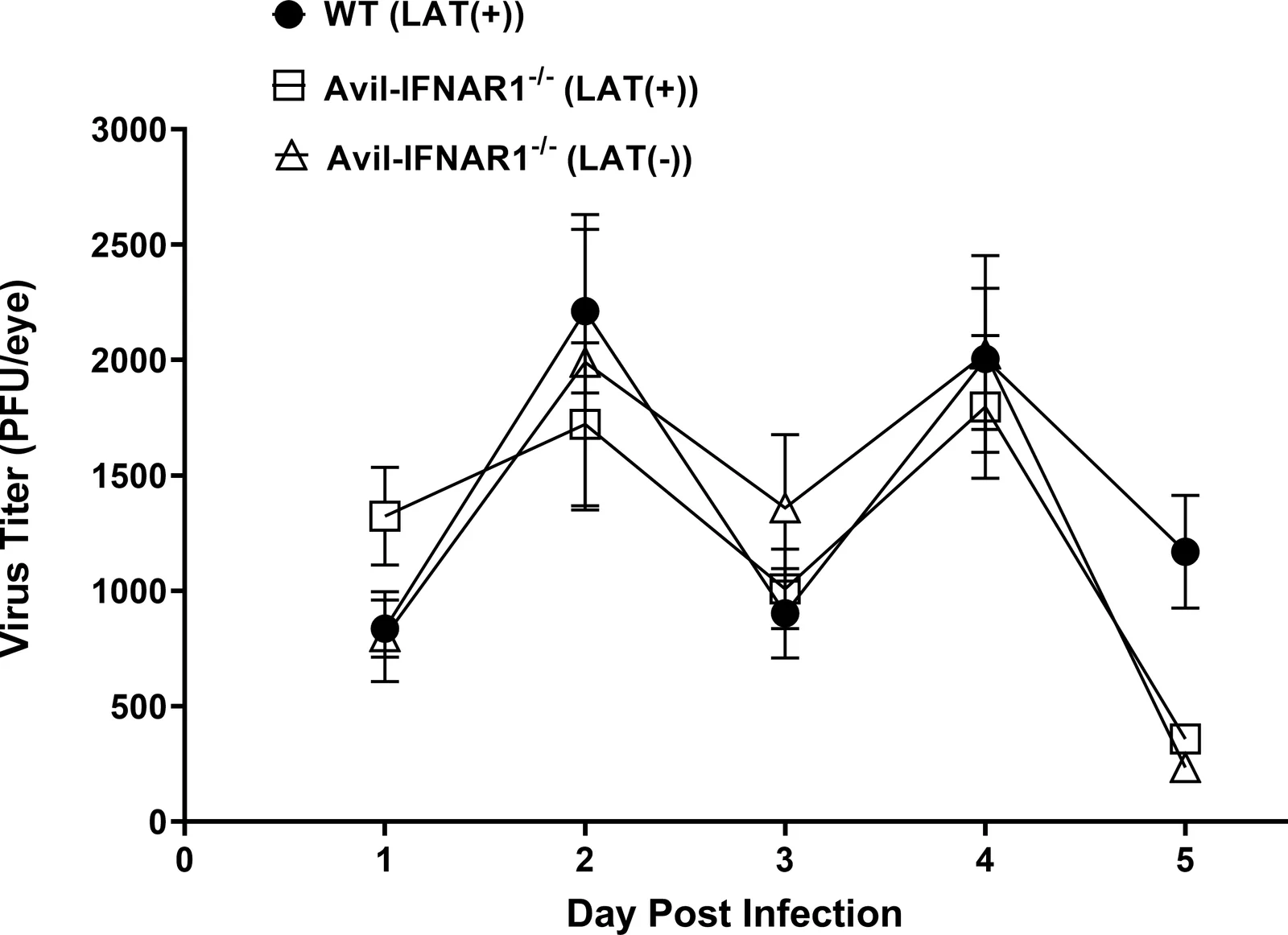
Virus titers in the tear films of infected mice. WT or Avil-IFNAR1^−/−^ mice were ocularly infected with LAT(+) or LAT(−) virus at a dose of 2 × 10^5^ pfu/eye. Tear films were collected on days 1−5 PI, and virus titers were measured by the standard plaque formation assay. Each point represents mean ± SEM titers of 40 eyes for WT mice infected with LAT(+), 42 eyes for Avil-IFNAR1^−/−^ mice infected with LAT(+), and 22 eyes for Avil-IFNAR1^−/−^ mice infected with LAT(−). Experiments were repeated twice.

We have previously reported that knockout of IFNα2A or IFNβ does not affect virus titers in tear films collected after primary infection (37, 38). To determine whether sensory neuron-specific knockout of IFNAR1 affects virus titers in tears, WT or Avil-IFNAR1^−/−^ mice were ocularly infected with either LAT(+) or LAT(−) virus. Tear films were collected on days 1–5 post-infection (PI), and virus titers were measured by standard plaque assay (Fig. 2). Virus titers were similar in all three groups, indicating that the absence of IFNAR1 in sensory neurons does not alter the amount of virus secreted into the tears on days 1–5 PI in either LAT(+)- or LAT(−)-infected mice.

### Absence of IFNAR1 in sensory neurons increases viral gene expression in the eye and in the TG of infected mice

**Fig 3:**
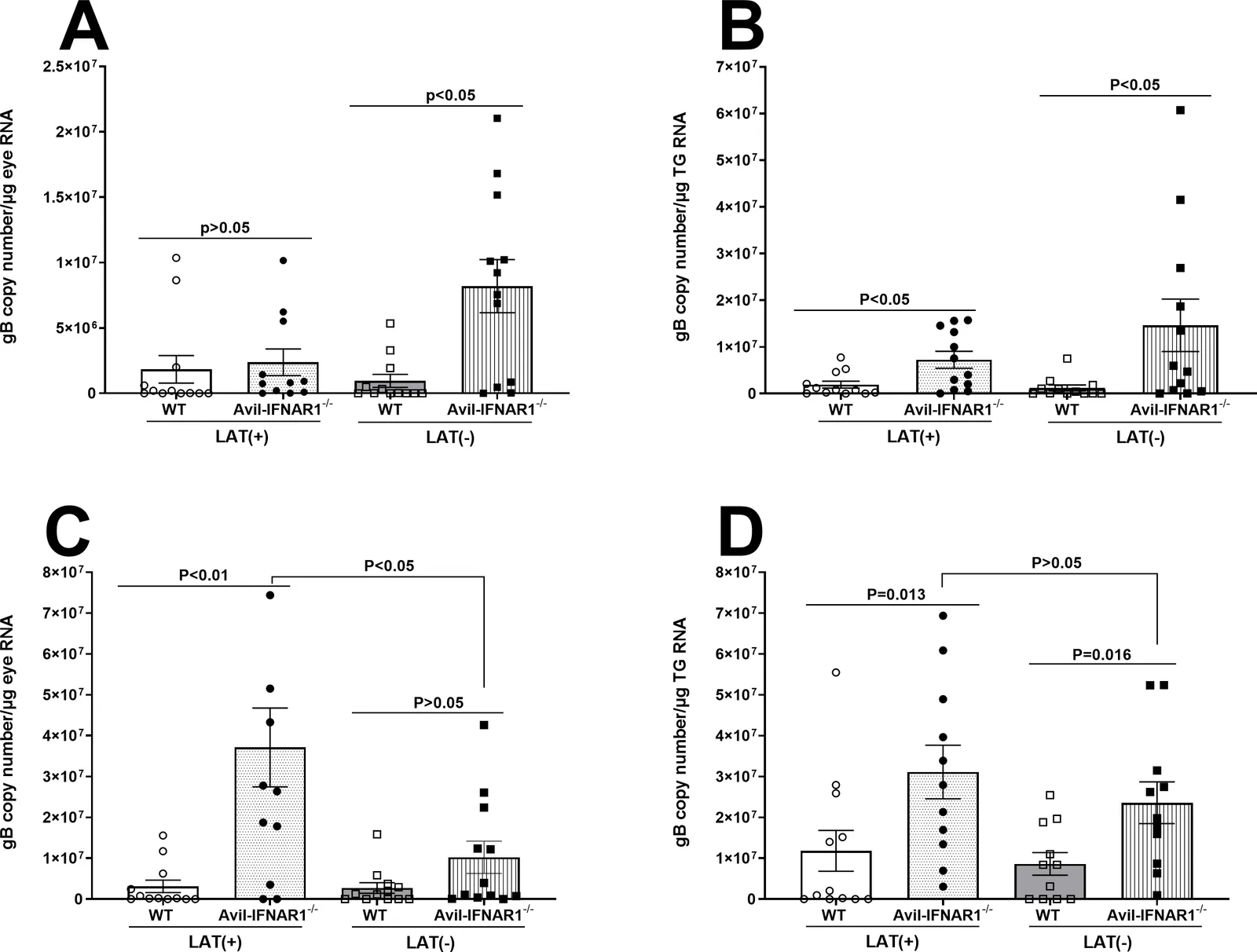
Effect of IFNAR1 absence on viral gene expression in the eye and TG during primary mouse infection. WT or Avil-IFNAR1^−/−^ mice were ocularly infected with 2 × 10^5^ pfu/eye of LAT(+) or LAT(−) viruses as in Fig. 1. Eyes (panels** A** and** C**) or TG (panels** B** and** D**) were harvested on days 3 and 5 PI. Total RNA was extracted, and gB copy number was determined by qRT-PCR and normalized to GAPDH. Each point represents the mean copy number ± SEM of 12 eyes and TG for each day.

To determine the effects of IFNAR1 knockout in sensory neurons on HSV-1 acute infection, WT or Avil-IFNAR1^−/−^ mice were ocularly infected with LAT(+) and LAT(−) viruses, and the eye (Fig. 3 A&C from eye) and TG (Fig. 3 B &D from TG) were harvested on days 3 and 5 PI. RNA isolated from each eye and TG was analyzed by TaqMan RT-PCR to determine gB copy numbers (Fig. 3). GAPDH mRNA in each sample was used as an internal control. The levels of gB transcripts in the eyes of WT and Avil-IFNAR1^−/−^ mice infected with LAT(+) virus on day 3 PI were not significantly different (Fig. 3A and *P* > 0.05, LAT(+)). However, Avil-IFNAR1^−/−^ mice infected with LAT(−) virus expressed significantly higher levels of gB than similarly infected WT mice (Fig. 3A and *P* < 0.05, LAT(−)). In contrast to the eye, TG of Avil-IFNAR1^−/−^ mice infected with either virus expressed significantly more gB than WT mice (Fig. 3B and *P* < 0.05).

On day 5 PI, levels of gB transcripts in the eye were significantly higher in LAT(+)-infected Avil-IFNAR1^−/−^ mice than in WT-infected mice (Fig. 3C, *P* < 0.01, LAT(+)). Despite higher gB expression in Avil-IFNAR1^−/−^ mice infected with LAT(−) virus than in similarly infected WT mice, this difference was not statistically significant (Fig. 3C and *P* > 0.05, LAT(−)). Finally, on day 5 PI, we observed significantly higher gB expression in the TG of Avil-IFNAR1^−/−^ mice infected with LAT(+) (Fig. 3D and *P* = 0.013) and LAT(−) (Fig. 3D and *P* = 0.016) viruses. Thus, loss of IFNAR1 in sensory neurons altered viral gene expression in the eyes and TG of infected mice, with more pronounced differences in the TG, suggesting that, in contrast to virus replication in tears of infected mice, type 1 IFN is important for viral gene expression during primary infection.

### Effect of IFNAR1 absence on mouse survival

**Fig 4:**
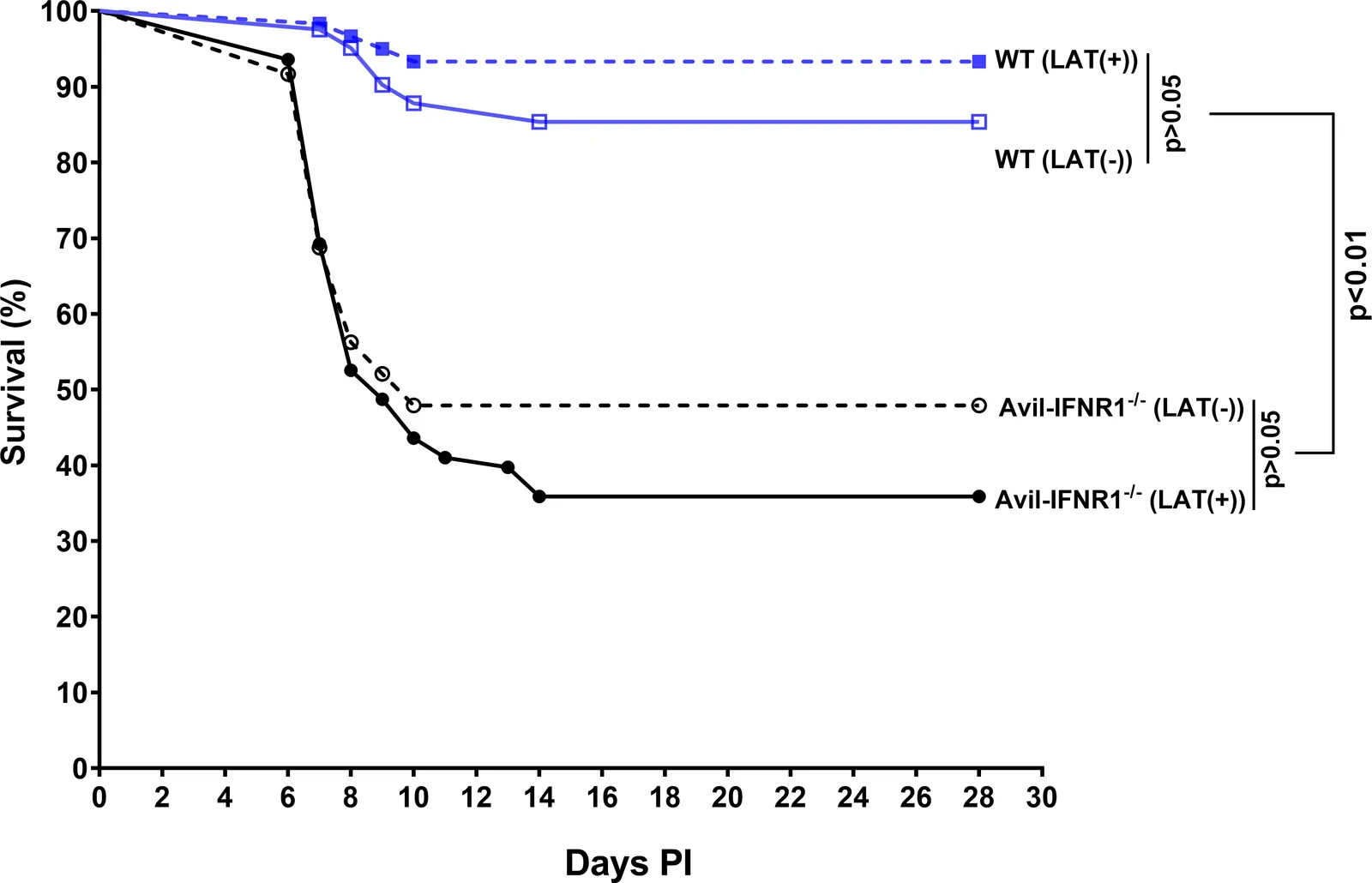
Survival of Avil-IFNAR1^−/−^ mice infected with LAT(+) and LAT(−) viruses. WT (blue lines) or Avil-IFNAR1^−/−^ mice (black lines) were ocularly infected with LAT(+) or LAT(−) virus at a dose of 2 × 10^5^ pfu/eye. Infected mouse survival was determined on day 28 PI, and the survival curve represents survival across four independent experiments.

We previously reported that IFNAR1^−/−^ mice are highly susceptible to HSV-1 infection, even when infected with as little as 100 pfu/eye of LAT(−) virus (36). Thus, we compared the survival of WT and Avil-IFNAR1^−/−^ mice after infection with 2 × 10^5^ pfu/eye of LAT(+) or LAT(−) virus (Fig. 4). Survival data are based on four independent experiments. In WT mice, 56 of 60 (93%) mice infected with LAT(+) virus survived ocular infection, while 28 of 78 (36%) of Avil-IFNAR1^−/−^ mice similarly infected with LAT(+) virus survived, and these differences were highly significant (Fig. 4 and *P* < 0.01, LAT(+)). Similar to LAT(+)-infected WT mice, 35 of 41 (85%) of WT mice infected with LAT(−) virus survived ocular infection, while 23 of 48 (48%) of Avil-IFNAR1^−/−^ mice similarly infected with LAT(−) virus survived, and these differences were highly significant (Fig. 4 and *P* < 0.01, LAT(−)). However, we did not find a significant difference in survival between LAT(+)- and LAT(−)-infected WT or Avil-IFNAR1^−/−^ mice (Fig. 4 and *P* > 0.05). Thus, the absence of IFNAR1 in infected mouse neurons decreased the survival of Avil-IFNAR1^−/−^ mice, independent of LAT presence.

### Absence of IFNAR1 in sensory neurons increases eye disease in infected mice

**Fig 5:**
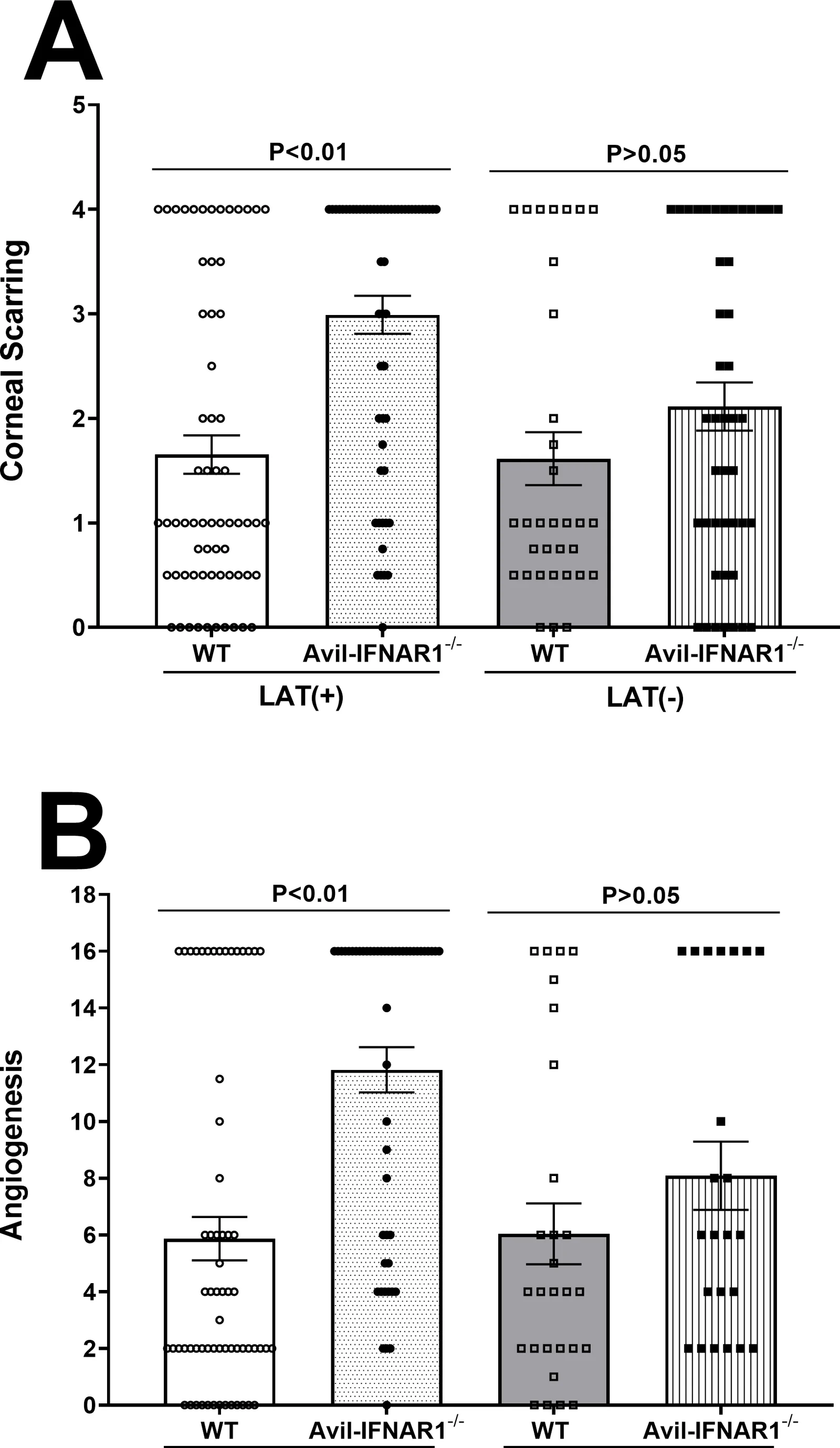
Effect of IFNAR1 absence in sensory neurons on HSV-1 induced eye diseases. The severity of CS and angiogenesis in surviving mice infected with LAT(+) or LAT(−) viruses was assessed on day 28 PI. Each bar represents the mean ± SEM from 112 eyes (open circles, WT, LAT(+)), 70 eyes (open squares, WT, LAT(−)), 56 eyes (closed circles, Avil-IFNAR1^−/−^, LAT(+)), and 46 eyes (closed squares, Avil-IFNAR1^−/−^, LAT(−)).

To compare the effect of IFNAR1 absence in sensory neurons of Avil-IFNAR1^−/−^ and WT mice after infection with LAT(+) and LAT(−) viruses, CS and angiogenesis in surviving mice were measured on day 28 PI (Fig. 5). Significantly more CS was observed in Avil-IFNAR1^−/−^ mice infected with LAT(+) virus than in WT-infected control mice (Fig. 5A and *P* < 0.01, LAT(+)). In contrast, the levels of CS in Avil-IFNAR1^−/−^ mice infected with LAT(−) did not differ significantly from levels in similarly infected WT mice (Fig. 5A and *P* > 0.05, LAT(−)). CS levels were similar in WT mice infected with either virus (Fig. 5A and WT, *P* > 0.05) and in Avil-IFNAR1^−/−^ mice infected with LAT(−) virus (Fig. 5A and Avil-IFNAR1^−/−^, *P* > 0.05). Similar to CS, Avil-IFNAR1^−/−^ mice infected with LAT(+) virus had significantly higher levels of angiogenesis (Fig. 5B and *P* < 0.01, LAT(+)) than WT mice infected with the same virus, while angiogenesis levels were similar in Avil-IFNAR1^−/−^ and WT mice infected with LAT(−) virus (Fig. 5B and *P* > 0.05, LAT(−)). Angiogenesis levels were similar in WT mice infected with either virus (Fig. 5B and WT, *P* > 0.05) and in Avil-IFNAR1^−/−^ mice infected with the LAT(−) virus (Fig. 5B and Avil-IFNAR1^−/−^, *P* > 0.05). These results suggest that the presence of LAT in Avil-IFNAR1^−/−^-infected mice is associated with increased eye disease relative to WT-infected mice and that this increased disease may be linked to subclinical reactivation in the presence of LAT and the absence of type 1 IFN.

### Effect of IFNAR1 absence in sensory neurons on HSV-1 latency

**Fig 6:**
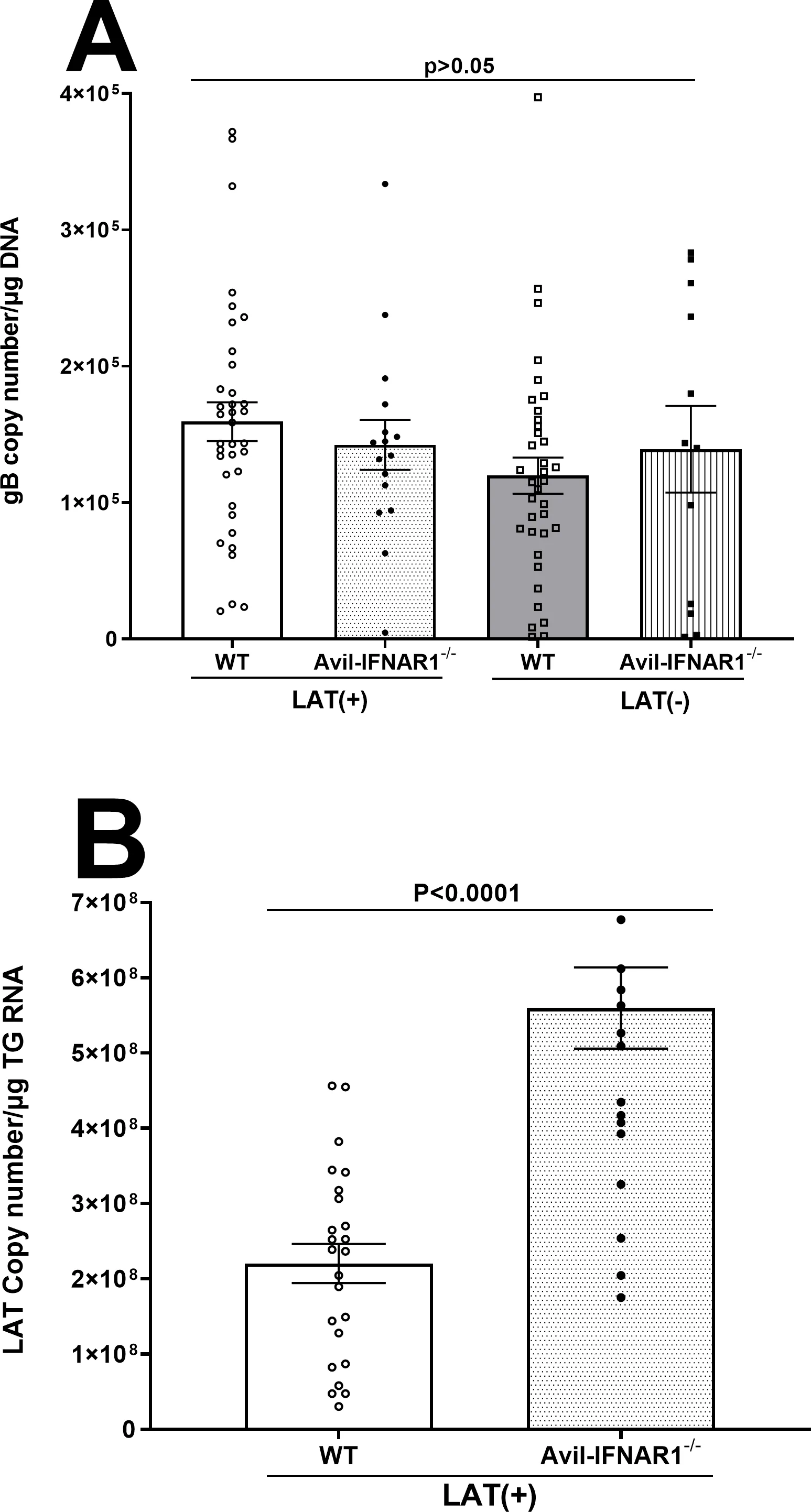
Levels of gB DNA and LAT in TG of latently infected mice. (**A**) gB DNA. WT or Avil-IFNAR1^−/−^ mice were ocularly infected with 2 × 10^5^ pfu/eye of LAT(+) or LAT(−) viruses. TG from surviving mice were harvested on day 28 PI, and total TG DNA was isolated as described in Materials and Methods. gB DNA copy number was measured by qPCR using a standard curve generated with pAc-gB1 DNA. GAPDH expression was used to normalize relative gB DNA expression levels. Each bar represents the mean ± SEM from 36 (WT, LAT(+)), 36 (WT, LAT(−)), 16 (Avil-IFNAR1^−/−^, LAT(+)), and 12 (Avil-IFNAR1^−/−^, LAT(−)) mice. (**B**) LAT in LAT(+)-infected mice. WT and Avil-IFNAR1^−/−^ mice were ocularly infected with 2 × 10^5^ pfu/eye of LAT(+) virus. TG was isolated on day 28, total RNA was extracted, and LAT copy numbers were calculated using a standard curve generated with pGem5317. Each bar represents the mean ± SEM from 24 TG for WT mice and 20 TG for Avil-IFNAR1^−/−^ mice from two independent experiments.

To determine the effect of sensory neuron-specific knockout of IFNAR1 on HSV-1 latency, WT or Avil-IFNAR1^−/−^ mice were infected with HSV-1 LAT(+) or LAT(−) virus, and TG were harvested on day 28 PI. Due to the absence of LAT in LAT(−)-infected mice, we isolated total DNA from individual TG from all four groups of infected mice, and TaqMan qPCR was performed to quantify viral gB DNA (see Materials and Methods). The amount of gB DNA in WT mice infected with LAT(+) virus and in Avil-IFNAR1^−/−^ mice infected with LAT(+) or LAT(−) viruses was similar (Fig. 6A and *P* > 0.05 for all four groups). Consistent with previous reports (14, 16, 40), WT mice infected with LAT(+) virus had significantly more gB DNA than WT mice infected with LAT(−) virus (Fig. 6A and *P* < 0.05, WT), suggesting that in the absence of type 1 IFN in Avil-IFNAR1^−/−^ mice, latency levels are not affected in the presence or absence of LAT in LAT(+) and LAT(−) viruses.

We next evaluated LAT expression in WT and Avil-IFNAR1^−/−^ mice infected with LAT(+) virus. WT and Avil-IFNAR1^−/−^ mice were infected with 2 × 10^5^ pfu/eye of LAT(+) virus as above. On day 28 PI, individual TG was harvested, and LAT copy numbers were measured by qRT-PCR. LAT transcript levels were significantly higher in TG from the Avil-IFNAR1^−/−^ group than in TG from the WT group (Fig. 6B and *P* < 0.0001). Our results showed that the absence of IFNAR1 in neurons of infected mice significantly increased LAT expression but did not affect gB levels, confirming the strong inhibitory role of type 1 IFN responses in sensory neurons. This result is consistent with a previous publication showing that type 1 interferon inhibits LAT expression (41). Our current results are similar to our previous studies, showing that levels of LAT expression in IFNβ^−/−^ mice infected with LAT(+) virus differ significantly from WT mice infected with LAT(+) virus, while no differences in levels of gB DNA were detected in the TG of Avil-IFNAR1^−/−^ and WT mice infected with LAT(+) and LAT(−) viruses (37).

### Absence of IFNAR1 in sensory neurons increases T-cell exhaustion but does not compensate for LAT effects on T-cell exhaustion

**Fig 7:**
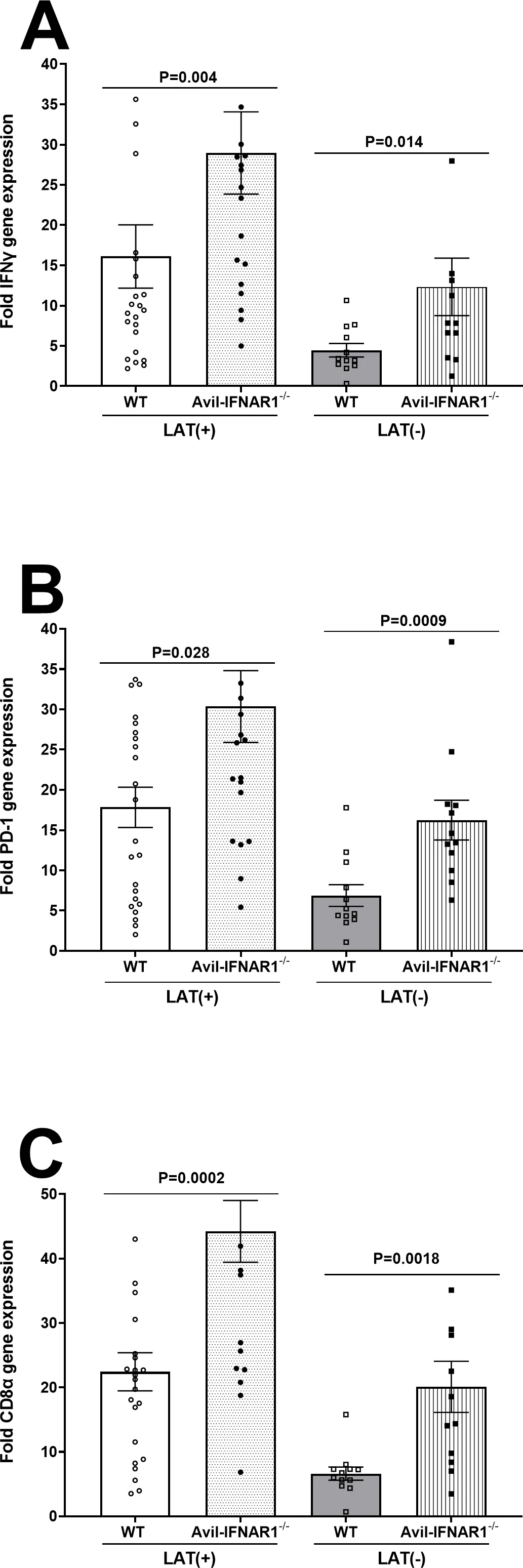
Effect of IFNAR1 absence on T-cell exhaustion in TG of latently infected mice. Avil-IFNAR1^−/−^ and WT mice were ocularly infected with 2 × 10^5^ pfu/eye of LAT(+) and LAT(−) viruses as above. TG from latently infected mice were individually isolated on day 28 PI, and qRT-PCR was performed using total RNA as described in Materials and Methods. IFNγ (panel **A**), PD-1 (panel **B**), and CD8α (panel **C**) expression in naive mice was used as a baseline control to estimate relative expression of each transcript in the TG of latently infected mice. GAPDH expression was used to normalize transcript expression. Each bar represents the mean ± SEM from 24 TG for WT mice infected with LAT(+), 12 TG for WT mice infected with LAT(−), 20 TG for Avil-IFNAR1^−/−^ mice infected with LAT(+), and 12 TG for Avil-IFNAR1^−/−^ mice infected with LAT(−).

LAT transcripts are known to increase T-cell exhaustion in latently infected mice (16, 42). T-cell exhaustion marker genes in the TG of LAT(+) virus-infected mice are known to be higher than in LAT(−) virus-infected mice (16). To determine how the absence of IFNAR1 in sensory neurons affects T-cell exhaustion, WT or Avil-IFNAR1^−/−^ mice were infected with LAT(+) or LAT(−) virus as above. TG from infected mice were harvested on day 28 PI, total TG RNA was isolated, and the relative levels of IFNγ, PD-1, and CD8α mRNA were determined by qRT-PCR of the total TG extracts. The results are presented as “fold” increase compared to baseline TG mRNA levels from uninfected their respective uninfected naive mice (Fig. 7). IFNγ expression in Avil-IFNAR1^−/−^ mice infected with LAT(+) virus was significantly higher than in similarly infected WT mice (Fig. 7A and *P* = 0.004, LAT(+)). IFNγ expression in Avil-IFNAR1^−/−^ mice infected with LAT(−) virus was also significantly higher than in WT mice infected with LAT(−) virus (Fig. 7A and *P* = 0.014). IFNγ expression was lower in LAT(−)-infected mice than in LAT(+)-infected mice (Fig. 7A and compare LAT(+) and LAT(−) groups).

Similar to IFNγ results, PD-1 expression in Avil-IFNAR1^−/−^ mice infected with LAT(+) virus was significantly higher than that in similarly infected WT mice (Fig. 7B and *P* = 0.028, LAT(+)). PD-1 expression was also higher in Avil-IFNAR1^−/−^ mice infected with LAT(−) virus than in infected WT LAT(−) mice (Fig. 7B and *P* = 0.0009, LAT(−)). In the absence of LAT in LAT(−)-infected mice, both WT and Avil-IFNAR1^−/−^-infected mice had significantly lower PD-1 expression (Fig. 7B and compare LAT(+) with LAT(−) group). Finally, CD8α expression in Avil-IFNAR1^−/−^ mice infected with LAT(+) virus (Fig. 7C and *P* = 0.0002, LAT(+)) or infected with LAT(−) virus (Fig. 7B and *P* = 0.0018, LAT(−)) was significantly higher than their WT counterparts. CD8α expression in LAT(−)-infected mice was significantly lower than in LAT(+)-infected mice (Fig. 7C and compare LAT(+) with LAT(−) groups).

These results suggest that the absence of IFNAR1 in sensory neurons contributes to higher T-cell exhaustion in the TG of latently infected mice, and the effect is more pronounced in LAT(−)-infected mice than in LAT(+)-infected mice. We previously reported that in mice lacking IFNβ expression, its absence compensates for the absence of LAT function in LAT(−)-infected mice, leading to increased PD-1 and CD8α expression, comparable to that seen in LAT(+) mice. In contrast, the absence of IFNAR1 in sensory neurons did not compensate for the loss of LAT function, as IFNβ expression was absent (37).

### Effects of IFNAR1 absence on reactivation in infected mice

**Fig 8:**
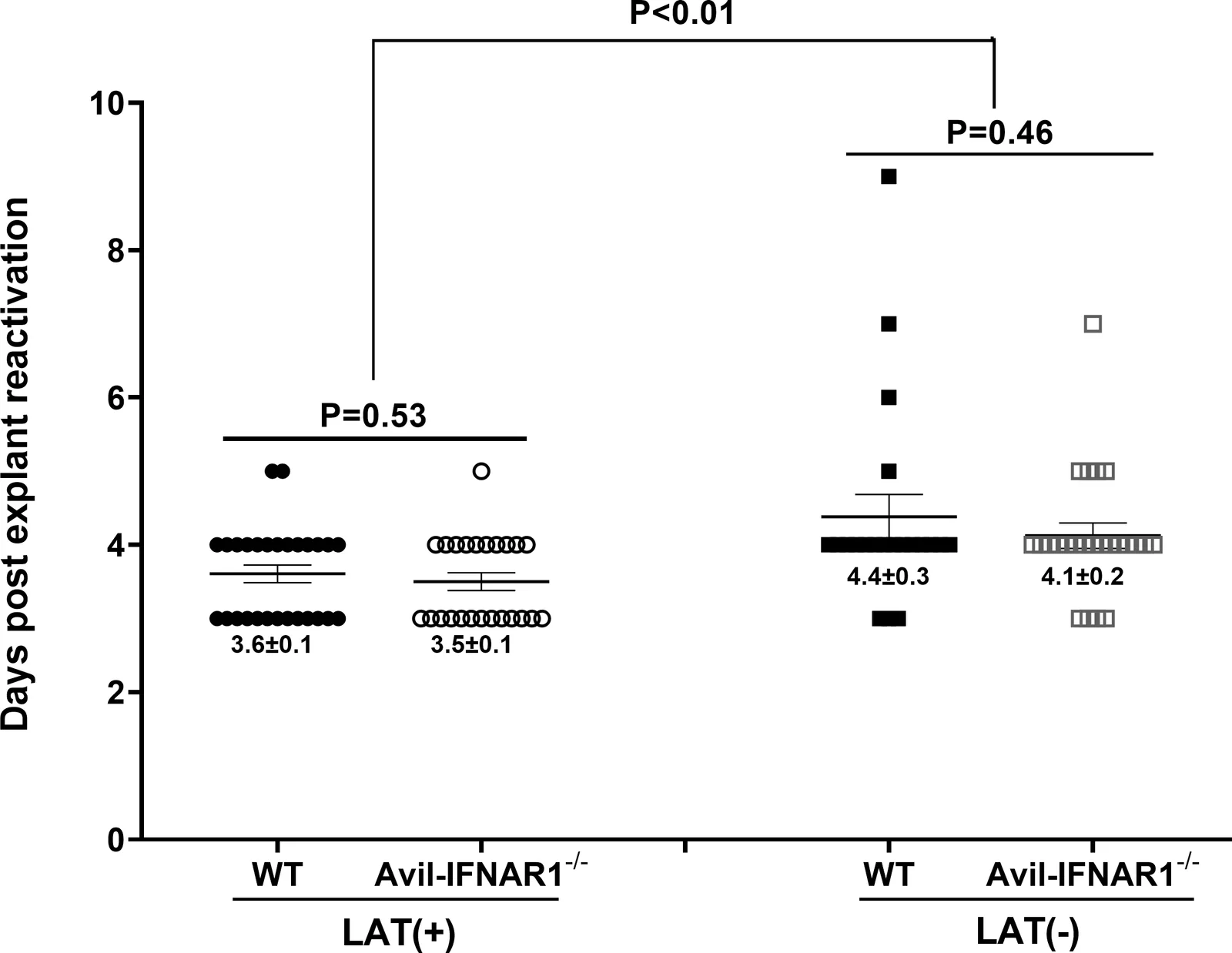
Effect of IFNAR1 absence on explant reactivation. Avil-IFNAR1^−/−^ and WT mice were ocularly infected with 2 × 10^5^ pfu/eye of LAT(+) and LAT(−) viruses as above. TG from infected mice were harvested on day 28 PI, and an explant reactivation assay was performed. Data points indicate the day of virus reactivation. Each point is the mean ± SEM from 28 TG for WT mice infected with LAT(+) virus, 21 TG for WT mice infected with LAT(−) virus, 24 TG for Avil-IFNAR1^−/−^ mice infected with LAT(+) virus, and 24 TG for Avil-IFNAR1^−/−^ mice infected with LAT(−) virus. The data represent two independent experiments.

Spontaneous reactivation in rabbits and induced reactivation both depend on the presence of LAT (14, 43). Therefore, we asked whether the absence of type 1 IFN in Avil-IFNAR1^−/−^ mice affects the time of reactivation. To test this, Avil-IFNAR1^−/−^ and WT mice were infected with 2 × 10^5^ pfu/eye of LAT(+) and LAT(−) viruses. TG was harvested from the four mouse groups on day 28 PI, and virus reactivation kinetics were measured in explanted TG. Average reactivation times for WT and Avil-IFNAR1^−/−^ mice infected with LAT(+) virus were 3.6 ± 0.1 days and 3.5 ± 0.1 days, respectively (Fig. 8 and *P* = 0.54, LAT(+)). Avil-IFNAR1^−/−^ mice infected with LAT(−) virus had an average reactivation time of 4.1 ± 0.2 days, while WT-infected mice had a reactivation time of 4.4 ± 0.3 days. These differences were not statistically significant (Fig. 8 and *P* = 0.46, LAT(−)). As expected, the time of reactivation in LAT(−) infected mice was slower than that in LAT(+) infected mice, and these differences were statistically significant (Fig. 8 and compare LAT(+) with LAT(−)). Taken together, our results suggest that the absence of IFNAR1 in sensory neurons does not alter the time of reactivation in either LAT(+) or LAT(−) viruses. This observation is intriguing because it suggests that type I IFN signaling in sensory neurons is not the dominant regulator of reactivation timing. This suggests that HSV-1 reactivation is controlled by a complex and redundant network of immune factors. The preservation of normal reactivation kinetics despite neuronal IFNAR1 deficiency suggests that alternative antiviral pathways, such as IFNγ signaling or other innate immune mechanisms, may compensate for the loss of type I interferon signaling during latency. Here, IFNγ signaling is shown to be independent of IFNα/β receptor components.

## DISCUSSION

When HSV-1 infects the host and establishes latency, the LAT transcript is the only viral gene abundantly expressed during the latent phase (2, 4). LAT was originally thought to be expressed exclusively in neurons during latency, but recent studies have shown LAT expression in other cell types, including immune cells (e.g., DCs and fibroblasts) at lower levels than in neurons (44, 45). As the major viral transcript during the latent phase, LAT has been extensively studied. Multiple LAT transcript functions have been reported, including maintaining latency, inhibiting apoptosis, and modulating lytic gene expression (14, 15, 43, 46, 47). LAT expression and enhancing latency both correlated with increased T-cell exhaustion (16, 48, 49). Replacing LAT with alternative anti-apoptotic genes restores the wild-type reactivation phenotype in a LAT(−) virus (17, 19). We have also shown that the absence of IFNβ can restore LAT function during LAT virus infection and restore reactivation and PD-1 expression to levels seen in LAT(+) virus (37). In this study, we showed that knockout of IFNAR1 in sensory neurons increases gB DNA copy number during LAT(−) infection to levels observed in LAT(+) virus, whereas we observed no significant difference between WT and Avil-IFNAR1^−/−^ mice infected with LAT(+) virus. Interestingly, in LAT(+) infected mice, we found significantly higher LAT expression in Avil-IFNAR1^−/−^ mice than in infected WT mice, despite similar gB DNA copy numbers between the two groups. Rosato et al. also reported similar HSV-1 genome copy numbers despite significantly higher LAT expression when they compared a neuron-specific Stat1 knockout with WT mice (41). We previously reported that DCs and macrophages isolated from Stat1 knockout mice were more susceptible to HSV-1 infection than those isolated from WT mice (50). The neuron-specific Stat1 knockout mice were highly susceptible to HSV-1 infection (41), which differs from our observation that approximately 40% of the infected Avil-IFNAR1^−/−^ mice survive. These differences may be due to the loss of all three IFN response types in neurons; Stat1 is the central transcription factor that transduces signals from all three types of IFN, whereas in our model, only type 1 IFN responses were deleted. Taken together, the current study shows that the type 1 IFN response is a key inhibitor of LAT expression in sensory neurons during latent infection.

Our previous report showed that LAT(+) viruses had significantly higher expression of T-cell exhaustion marker genes than LAT(−) viruses (16, 50, 51). In this study, we confirmed that WT and Avil-IFNAR1^−/−^ mice infected with LAT(+) virus expressed significantly more IFNγ, PD-1, and CD8α than did similarly infected LAT(−) mice. LAT(+)- and LAT(−)-infected mice both expressed higher levels of IFNγ, PD-1, and CD8α in the absence of IFNAR1 than in WT mice, suggesting that a possible parallel LAT and type 1 IFN pathway in sensory neurons controls T-cell exhaustion.

We previously reported that LAT suppresses IFNα responses in the TG of infected mice (13, 52, 53). In mice, the IFNα family is encoded in 14 genes (30, 32–34, 54). We generated a unique mouse knockout of one of these 14 IFNα genes, IFNα2A (Roferon-A) (38). We found that LAT expression, but not reactivation, was significantly reduced in infected IFNα2A^−/−^ mice (38). We also reported that mice are more susceptible to HSV-1 infection in the absence of IFNβ, causing more severe eye disease. The absence of IFNβ can partially compensate for LAT gene function, increase latency in LAT(−) virus to levels seen in LAT(+) virus, and accelerate *in vitro* reactivation of LAT(−) virus to levels seen in LAT(+) virus (37). In contrast to global knockout of IFNAR1, mice that are highly susceptible to infection even at 1 × 10^3^ pfu/eye of HSV-1, especially in LAT(−)-infected mice (36), while Avil-IFNAR1^−/−^ mice were less susceptible to HSV-1 infection even at a 100-fold higher viral dose, regardless of the presence or absence of LAT.

There are no reports that LAT is directly involved in pathological eye diseases in infected mice. We previously reported no significant difference in eye disease in mice infected with LAT(+) or LAT(−) viruses (55). As in previous studies, we did not see any differences in the incidence of eye diseases between LAT(+) and LAT(−) virus infections in WT mice. However, Avil-IFNAR1^−/−^ mice infected with LAT(+), but not LAT(−), virus had significantly higher levels of CS and angiogenesis than WT mice. These results suggest an important role of the LAT transcript in the development of pathological eye diseases, which can be inhibited by type 1 IFN responses in WT mice. Similar to IFNα2A^−/−^ and IFNβ^−/−^ mice (37, 38), virus titers in the tears of infected mice were not affected by the absence of IFNAR1 in infected mouse neurons. This is consistent with our previous studies, in which deleting the signal peptide peptidase in the eye affected virus replication in the eye, but not in TG. However, deleting signal peptide peptidase in the TG affected virus replication in the TG but not in the eye (39, 56). Despite finding no differences in virus titers in tears, gB expression levels in eyes and TG were significantly higher in Avil-IFNAR1^−/−^ mice than in the control WT mice at both days 3 and 5 PI. These results confirm that the type I IFN response plays a critical role in viral defense in neurons. Of note, viral gene expression was significantly higher in eyes from LAT(+) virus infected Avil-IFNAR1^−/−^ mice than in LAT(−) infected Avil-IFNAR1^−/−^ mice or WT infected mice. The higher levels of viral transcripts observed in the eye and TG of Avil-IFNAR1^−/−^ mice infected with LAT(+) could explain the greater eye disease observed in this group, despite similar reactivation times in Avil-IFNAR1^−/−^ and WT mice. Thus, the absence of type I IFN responses in the TG may have contributed to increased retrograde-anterograde virus replication in the eye and TG of infected mice (7, 39).

In summary, sensory neuron-specific knockout of IFNAR1 increased viral replication in both the eye and TG during primary infection and increased LAT and exhaustion markers during latency, with LAT(+) virus causing more severe eye disease. Our results suggest a complex interplay between LAT and type 1 IFN responses in sensory neurons.

## MATERIALS AND METHODS

### Cells and viruses

The rabbit skin (RS) cell line was cultured in minimal essential medium (MEM) supplemented with 5% fetal bovine serum and maintained as described previously (53). Triple-plaque-purified HSV-1 strain McKrae (LAT(+)) and dLAT2903 (LAT(−)) were grown in RS cell monolayers as described previously (53). HSV strains McKrae and dLAT2903 are virulent and efficiently infect mice without corneal scarification. McKrae is the parental virus for dLAT2903 (14).

### Generation of Avil-IFNAR1^−/−^ mice

Avil-IFNAR1^−/−^ (IFNAR1^flox/flox^ Avil-Cre^+^) mice on a C57BL/6 background were generated in-house by breeding the IFNAR1^flox/flox^ mice (Jackson Laboratory #028256) with Avil-Cre^+^ mice (Jackson Laboratory #032536). Only male IFNAR1^flox/flox^ Avil-Cre^+^ mice were used as Avil-Cre donors to avoid germline Cre leakage in female mice. WT C57BL/6 mice were purchased from The Jackson Laboratory (Bar Harbor, ME), bred in-house at Cedars-Sinai Medical Center, and used as controls. In all of our studies, we used both male and female mice, and we did not detect any differences between them.

### Mouse genotyping

The following primers were used to screen Avil-IFNAR1^−/−^ mice: Forward, ACT CAG GTT CGC TCC ATC AG, and Reverse, CTT TTA ACC ACT TCG CCT CGT, producing a 325-bp PCR product for the mutant allele and a 395-bp PCR product for the wild-type allele (57). Primers used to screen Avil-Cre mice were the following: Forward, GCG ATC CCT GAA CAT GTC CAT C, and Reverse, CCC TGT TCA CTG TGA GTA GG (reverse), producing a 180-bp PCR product (58).

### Immunofluorescence staining

TG from 7- to 8-week-old WT control or Avil-IFNAR1^−/−^ mice were harvested and frozen in O.C.T. compound (Tissue-Tek). Frozen tissue was sectioned using an NX70 Cryostat. Tissue sections were fixed in 4% paraformaldehyde, washed in 1× phosphate-buffered saline (PBS), and then permeabilized with 0.3% Triton X-100 in PBS. Slides were blocked using 1× sea blocker (Thermo Scientific, Rockford, IL, USA) plus 5% donkey serum for 1 h at 25°C and then incubated with anti-IFNAR1 antibody (Ab) (Invitrogen, MA5-32006) in blocking buffer at 4°C overnight. Slides were washed thrice with 1× PBS and then incubated with secondary Ab (AlexaFluor 647 donkey anti-rabbit Ab) and AlexaFluor 488-conjugated anti-Tubulin β3 Ab in 1× blocking buffer for 2 h at room temperature. Slides were washed four times with 1× PBS and mounted in Prolong Gold antifade reagent with DAPI (Invitrogen, P86931). Image acquisition and data analysis were performed using a Leica DM4000 microscope (Leica Microsystems, Buffalo Grove, IL, USA).

### Ocular infection

WT control and Avil-IFNAR1^−/−^ mice (7–8 weeks old, both sexes) were ocularly infected in both eyes with 2 × 10^5^ pfu/eye of McKrae (LAT(+)) or dLAT2903 (LAT(−)) in MEM as an eye drop without corneal scarification, as we described previously (59).

### Viral titers from tears of infected mice

Tear films were collected from the eyes of infected mice each day from days 1 to 5 PI using a cotton applicator swab. Each swab was placed in 1 mL of MEM and stored at −80°C until titration. Viral titer was determined using a standard plaque assay on RS cells (53).

### Measurement of mouse eye diseases

The severity of CS lesions in mouse corneas was assessed by slit-lamp biomicroscopy using a double-blind approach, with the investigator performing scoring while blinded to experimental group assignments. The scoring scale was as follows: 0, normal cornea; 1, mild haze; 2, moderate opacity; 3, severe corneal opacity but iris visible; and 4, opaque, corneal ulcer, and corneal rupture, as described previously (60). Angiogenesis was scored using a system in which a grade of 4 for a given quadrant of the circle represents a centripetal growth of 1.5 mm toward the corneal center. The four-quadrant scores were summed to derive the neovessel index (range: 0–16) for each eye.

### *In vitro* explant reactivation assay

TG from infected mice were harvested 28 days PI, cultured in 1.5 mL of MEM, and reactivation assays were performed as described previously (53). Briefly, a 100-μL aliquot was collected from each culture daily and used to infect RS cell monolayers, which were monitored daily for 3 days for the appearance of cytopathic effect to determine when reactivated virus first appeared from each TG. Media from explanted TG cultures were plated daily, and the time at which reactivated virus first appeared was determined.

### Determining the levels of gB DNA and LAT RNA in TG of latently infected mice

To detect latent virus copy number, TG from individual mice were collected on day 28 PI, and genomic DNA was isolated using a Qiagen tissue DNA isolation kit (Qiagen). gB DNA levels from latent TG were determined using custom-made gB primers and probe as follows: Forward: 5′-AAC GCG ACG CAC ATC AAG-3′; Reverse: 5′-CTG GTA CGC GAT CAG AAA GC-3′; and Probe: 5′ FAM-CAG CCG CAG TAC TAC C-3′ (where FAM is 6-carboxyfluorescein) (amplicon length = 72 bp). Estimated relative gB copy number was calculated using standard curves generated from plasmid pAc-gB1 (61). Briefly, the plasmid DNA template was serially diluted 10-fold so that 1 μL contained 10^3^–10^8^ copies of the gB gene and then subjected to TaqMan PCR using the same primer set as test samples. To measure LAT expression in mice infected with LAT(+) virus, isolated TG were immersed in an RNA stabilization reagent (RNAlater, Thermo Fisher Scientific, Waltham, MA, USA) and stored at −80°C for processing. Total RNA was extracted, as we described previously (62). LAT RNA levels from latent TG were determined using the following custom LAT primers and probe: Forward: 5′-GGG TGG GCT CGT GTT ACA G-3′; Reverse: 5′-GGA CGG GTA AGT AAC AGA GTC TCT A-3′; and Probe: 5′-FAM-ACA CCA GCC CGT TCTT T-3′ (amplicon length = 81 bp). LAT RNA copy number was calculated using a standard curve generated using pGem5317 containing the LAT region, as previously described (62). In all experiments, GAPDH was used as an internal control. Copy number for each reaction product was determined by comparing the normalized threshold cycle (CT) of each sample to the threshold cycle of the standard curve.

### RT-qPCR to measure gene expression

TG or eyes from individual infected mice were isolated at the indicated times. Collected tissues were immersed in TRIzol reagent and stored at −80°C for processing. TG or eyes from each animal were processed for RNA extraction as described previously (37). Isolated total RNA was reverse transcribed using a high-capacity cDNA reverse transcription kit (Applied Biosystems, Foster City, CA, USA) according to the manufacturer’s protocol. Gene transcript expression levels were evaluated using TaqMan primer sets as follows: (i) CD8α (Mm01182107_g1), (ii) IFNγ (Mm01168134_m1), (iii) PD-1 (Mm00435532_m1), (iv) IFNβ (Mm00439552_s1), and (v) GAPDH (m999999.15_G1). GAPDH was used as an internal control. To analyze fold change in expression, the 2^−ΔΔCT^ method was used to calculate the fold change relative to expression in uninfected controls in each group. Statistical differences were calculated using the ΔCT value.

### Statistical analysis

Student’s *t*-test was performed using the computer program Instat (GraphPad, San Diego, CA, USA). The results were considered statistically significant at a *P*-value of <0.05. All experiments were repeated at least twice to ensure accuracy. For RT-PCR analysis, statistical significance was assessed using ΔCT, with *P* < 0.05 considered significant. Error bars for each figure were automatically calculated by GraphPad Prism software, based on the individual fold change value 2^− ΔΔCT^.

## Data Availability

Data presented here, including raw images used for preparation of composite figures and data tables used for preparation of graphs, are available from the corresponding author upon request.

## SUPPLEMENTAL MATERIAL

Fig S1 jvi.00960-26-s0001.pptx Quantification of IFNAR1 immunostaining shown in Fig. 1B.

ASM does not own the copyrights to Supplemental Material that may be linked to, or accessed through, an article. The authors have granted ASM a non-exclusive, world-wide license to publish the Supplemental Material files. Please contact the corresponding author directly for reuse.
